# Smoking alters the antigenicity and infectivity of Porphyromonas gingivalis

**DOI:** 10.1186/1617-9625-12-S1-A14

**Published:** 2014-06-06

**Authors:** Iris Zeller, Justin A Hutcherson, Richard J Lamont, Donald R Demuth, Pinar Gumus, Nejat Nizam, Nurcan Buduneli, David A Scott

**Affiliations:** 1 Oral Health and Systemic Disease, University of Louisville, Louisville, Kentucky, 40292, USA; 2 School of Dentistry, University of Louisville, Louisville, Kentucky, 40292, USA; 3 Department of Periodontology, School of Dentistry, Ege University, Izmir, 35040, Turkey

## Background

Cigarette smokers are more susceptible to periodontal diseases and are more likely to be infected with Porphyromonas gingivalis than non-smokers. Furthermore, smoking is known to alter the expression of P. gingivalis surface components and to compromise IgG generation. The aim of this study was to evaluate if the overall IgG response to P. gingivalis is suppressed in smokers in vivo and if previously established in vitro tobacco-induced phenotypic P. gingivalis changes would be reflected in vivo.

## Materials and methods

We examined the humoral response to several P. gingivalis strains as well as specific tobacco-regulated outer membrane proteins (FimA and RagB) by ELISA in biochemically-validated (salivary cotinine) smokers and non-smokers with chronic (CP, n = 13) or aggressive (AP, n = 20) periodontitis. We also monitored the local and systemic presence of P. gingivalis DNA by PCR.

## Results

Smoking was associated with decreased total IgG responses against clinical (10512, 5607, and 10208C; all p < 0.05) but not laboratory (ATCC 33277, W83) P. gingivalis strains. Smoking did not influence IgG produced against specific cell surface proteins, although a non-significant pattern towards increased total FimA-specific IgG in CP subjects, but not AP subjects, was observed. Seropositive smokers were more likely to be infected orally and systemically with P. gingivalis (p < 0.001), as determined by 16S RNA analysis.

## Conclusions

Smoking alters the humoral response against P. gingivalis and may increase P. gingivalis infectivity, strengthening the evidence that mechanisms of periodontal disease progression in smokers may differ from non-smokers with the same disease classification.

